# Sarcopenia in type 2 diabetes mellitus: from muscle quantity to multidimensional muscle health

**DOI:** 10.3389/fendo.2026.1980238

**Published:** 2026-10-20

**Authors:** Zicheng Wang, Lilu Zhu, Fangli Zhang, Zhao Liu, Jun Lu, Tao Lei, Wenjun Sha

**Affiliations:** Department of Endocrinology, Putuo Hospital, Shanghai University of Traditional Chinese Medicine, Shanghai, China

**Keywords:** continuous glucose monitoring, muscle health, muscle quality, myosteatosis, sarcopenia, sarcopenic obesity, skeletal muscle, type 2 diabetes mellitus

## Abstract

Type 2 diabetes mellitus (T2DM) is increasingly recognized as a condition in which skeletal muscle impairment extends beyond loss of muscle mass alone. Epidemiologic evidence indicates that sarcopenia is more common in individuals with T2DM and is associated with mortality, cardiovascular disease, and diabetes-related complications. Recent research has therefore shifted toward a multidimensional view of muscle health that incorporates muscle strength, physical performance, muscle composition, myosteatosis, muscle density, and strength relative to muscle mass. This review synthesizes the epidemiology, clinical significance, diagnostic frameworks, and pathophysiological basis of sarcopenia in T2DM, with particular attention to the relationships among muscle mass, strength, composition, and function. It evaluates emerging evidence on myosteatosis, multimodal muscle assessment, continuous glucose monitoring-derived glycemic phenotypes, sarcopenic obesity, and contemporary glucose-lowering and weight-loss therapies. Current evidence remains limited by heterogeneous sarcopenia definitions, muscle-mass normalization methods, and measurement techniques, while studies of myosteatosis, glycemic variability, and emerging biomarkers are still predominantly observational. In addition, trials of potent glucose-lowering and weight-loss therapies commonly emphasize body weight, lean mass, or muscle volume rather than integrated muscle-centered outcomes. A more coherent framework that jointly evaluates muscle quantity, composition, strength, and physical performance is needed to improve early detection, risk stratification, and intervention research in diabetes-related muscle health.

## Graphical Abstract

**Figure:**
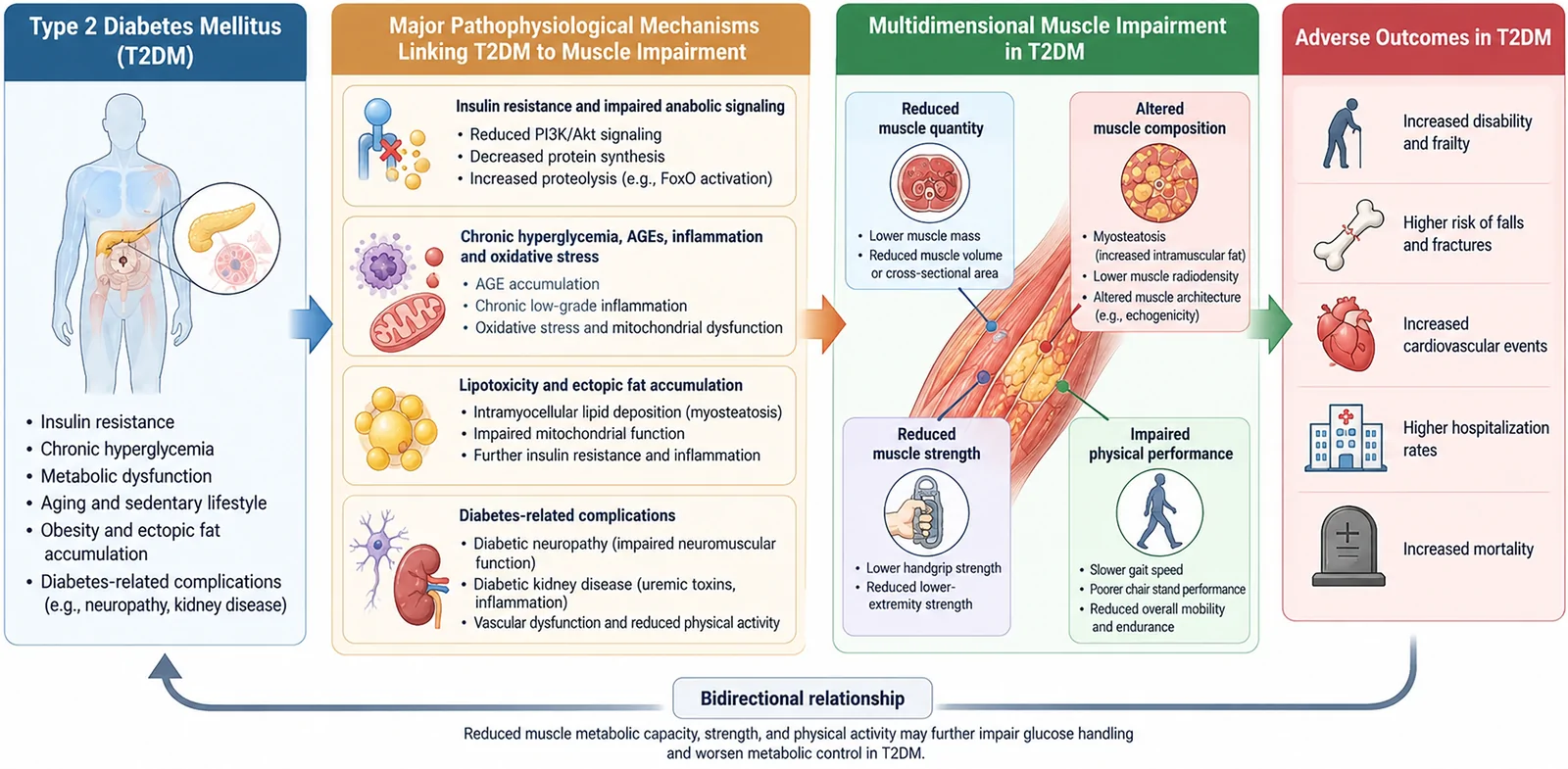

## 1 Introduction

Type 2 diabetes mellitus (T2DM) is characterized by insulin resistance, progressive β-cell dysfunction, and chronic hyperglycemia (1). Long-term management has traditionally focused on glycemic control and the prevention of cardiovascular, renal, and neurologic complications. As populations age and survival with T2DM improves, however, skeletal muscle impairment has emerged as an increasingly relevant determinant of mobility, quality of life, and long-term prognosis. Skeletal muscle is not only essential for locomotion and postural stability but is also a major site of insulin-mediated glucose disposal. Disturbances in glucose metabolism and deterioration in muscle function may therefore reinforce one another through a reciprocal metabolic-functional cycle (2).

Sarcopenia is a skeletal muscle disorder characterized by deficits in muscle quantity and strength, with physical performance contributing to the assessment of disease severity or clinical consequences depending on the diagnostic framework (3–6). Although the prioritization and operational roles of these components differ across consensus frameworks, sarcopenia is increasingly recognized as an important comorbidity of T2DM. Meta-analytic evidence indicates that individuals with T2DM have a higher prevalence and greater odds of sarcopenia than those with normal glucose metabolism, while sarcopenia in T2DM is associated with adverse outcomes including mortality, cardiovascular disease, and diabetes-related complications (7–9). These observations have increased interest in skeletal muscle health as a clinically relevant dimension of T2DM beyond conventional glycemic and cardiorenal outcomes.

Importantly, diabetes-related muscle impairment is not fully captured by muscle quantity alone. Reduced muscle strength and adverse muscle composition may occur even when absolute muscle quantity is relatively preserved (10, 11).

Recent consensus statements reflect a similar conceptual shift toward multidimensional muscle health. AWGS 2025 emphasizes the combined consideration of muscle quantity, muscle strength, and physical performance and extends attention to muscle health across a broader adult age range (12). This perspective is particularly relevant to T2DM, in which metabolic and chronic disease-related factors may affect muscle structure and function before advanced age.

Advances in metabolic monitoring, muscle imaging, and weight-loss pharmacotherapy have further broadened the study of muscle health in T2DM. CGM-derived glycemic metrics are being investigated in relation to muscle phenotypes (13–16), while contemporary glucose-lowering and weight-loss therapies have raised questions regarding how changes in lean mass, muscle volume, composition, and function should be interpreted during treatment (17). These developments reinforce the need to distinguish changes in individual muscle-related measures from clinically defined sarcopenia.

Despite these advances, interpretation of the literature remains limited by heterogeneous sarcopenia definitions, inconsistent measurement approaches, and predominantly observational evidence for several emerging muscle phenotypes. Moreover, muscle quantity, strength, physical performance, and composition are not consistently assessed together. A clearer multidimensional framework is therefore needed to interpret muscle health in T2DM.

This review synthesizes the epidemiology, clinical significance, diagnostic frameworks, and pathophysiological basis of sarcopenia in T2DM, with particular attention to the relationships and distinctions among muscle quantity, strength, physical performance, and composition. We critically evaluate emerging evidence on myosteatosis, multimodal muscle assessment, CGM-derived glycemic phenotypes, sarcopenic obesity, and contemporary glucose-lowering and weight-loss therapies. We also identify major limitations of the existing evidence base and outline priorities for earlier detection, risk stratification, and intervention research in diabetes-related muscle health.

## 2 Methods

This narrative review was developed through a literature search conducted between July 10 and August 26, 2026. The databases searched were PubMed, Web of Science Core Collection, China National Knowledge Infrastructure (CNKI), Wanfang Medical, SinoMed, and VIP Database. The search focused on combinations of terms related to type 2 diabetes mellitus, sarcopenia, skeletal muscle, muscle mass or quantity, muscle strength, physical performance, muscle composition, myosteatosis, diagnostic and imaging methods, continuous glucose monitoring, sarcopenic obesity, exercise, nutrition, and glucose-lowering or weight-loss therapies. Reference lists of relevant reviews, consensus statements, and key original studies were also examined to identify additional pertinent publications.

Given the narrative nature of this review, studies were selected according to their relevance to the major domains of diabetes-related muscle health rather than through formal systematic-review eligibility criteria. Priority was given to consensus statements for definitions and diagnostic frameworks, systematic reviews and meta-analyses for epidemiologic estimates and overall associations, longitudinal studies for temporal relationships and clinical outcomes, and randomized controlled trials or related body-composition analyses for intervention effects. Cross-sectional studies were included when they provided clinically relevant information on emerging phenotypes or assessment approaches. Narrative reviews were used primarily to provide conceptual and mechanistic context, with key quantitative claims preferentially supported by primary studies or higher-level evidence. Evidence was interpreted according to study design, with cross-sectional associations distinguished from longitudinal temporal relationships, intervention effects, and mechanistic evidence. The literature selection was intended to provide a representative and clinically relevant synthesis rather than an exhaustive systematic identification of all eligible studies.

The pharmacologic discussion was intentionally selective rather than comprehensive. SGLT2 inhibitors, GLP-1 receptor agonists, and tirzepatide were emphasized because they are directly relevant to the review’s focus on treatment-associated changes in body weight, lean tissue, muscle volume or composition, and their interpretation in relation to multidimensional muscle health. The muscle-related interpretation of contemporary interventions, including exercise, nutrition, and glucose-lowering or weight-loss therapies, is summarized in **Table 1**. Other glucose-lowering therapies were not systematically reviewed because the aim was not to provide a class-by-class appraisal of all antihyperglycemic agents; their omission should not be interpreted as evidence of absent muscle-related effects or clinical relevance.

**Table 1:** Muscle-relevant interpretation of contemporary interventions discussed in the review.

Intervention	Evidence summarized in this review	Muscle-related findings	Clinical interpretation	Key evidence gap
Resistance training	Meta-analysis of 43 randomized controlled trials; 2,012 adults aged ≥50 years with T2DM (18).	HbA1c −0.55 percentage points; HOMA-IR −1.15; fasting glucose −6.99 mg/dL; muscle mass +0.89 kg; upper- and lower-extremity strength SMDs +2.28 and +2.02.	Provides the most direct evidence for simultaneous metabolic and muscle benefit and should remain a foundation of muscle-preserving care.	Exercise protocols and outcome definitions vary; trials should report mass, strength, and physical performance together.
Nutrition	Evidence-based protein recommendations in older adults and the individualized approach discussed in this review (19).	Adequate energy and protein intake supports muscle protein synthesis, particularly when combined with resistance exercise.	Nutrition should be tailored to renal function, nutritional status, obesity, and the pace of weight loss rather than applying a uniform high-protein prescription.	T2DM-specific intervention trials using integrated sarcopenia and functional outcomes remain limited.
SGLT2 inhibitors	Meta-analysis of 25 randomized controlled trials; 2,286 participants with T2DM (20).	Lean mass −0.80 kg; skeletal muscle mass −0.38 kg; body water −0.96 kg, alongside reductions in body weight and fat mass.	Body-composition changes do not establish drug-induced sarcopenia; muscle status is best interpreted in the context of function and hydration.	BIA-derived estimates may be affected by fluid loss; strength and physical-performance data are sparse and sarcopenia was rarely prespecified.
GLP-1 receptor agonists	Systematic review/meta-analysis of 18 studies (21), interpreted in the context of expected lean-mass loss during substantial weight reduction (22–24).	Human studies report changes in lean mass or skeletal muscle index, but evidence on strength, physical performance, and rigorously defined sarcopenia is limited.	Lean-mass loss should be interpreted relative to total weight/fat loss and together with strength, performance, and tissue composition.	No clear human evidence establishes direct muscle injury or protection; older, frail, and low-muscle-reserve populations require dedicated study.
Tirzepatide	SURPASS-3 MRI *post hoc* analysis; 246 participants with evaluable MRI data at 52 weeks (17).	Thigh muscle volume −0.64 L and muscle fat infiltration −0.36 percentage points; volume loss was close to that predicted by weight loss, whereas fat infiltration improved more than predicted.	Illustrates that muscle quantity and composition can change in different directions; muscle-volume loss alone is insufficient to infer muscle injury.	*Post hoc*, industry-funded substudy; sarcopenia, muscle strength, and physical performance were not primary outcomes and prevention of sarcopenia was not demonstrated.

BIA, bioelectrical impedance analysis; GLP-1, glucagon-like peptide-1; HOMA-IR, homeostatic model assessment of insulin resistance; MRI, magnetic resonance imaging; SGLT2, sodium–glucose cotransporter 2; SMD, standardized mean difference; T2DM, type 2 diabetes mellitus.

## 3 Epidemiology and clinical burden

### 3.1 Conceptual evolution, terminology, and diagnostic frameworks

The concept of sarcopenia has evolved from an emphasis on reduced muscle mass toward a multidimensional construct that incorporates strength and function (25). Kirk et al. (2024) (26) on behalf of the Global Leadership Initiative in Sarcopenia (GLIS), conducted two rounds of an international Delphi process involving 107 experts from 29 countries and regions. Consensus was reached on muscle mass, muscle strength, and muscle-specific strength as conceptual components of sarcopenia, with agreement rates of 89.4%, 93.1%, and 80.8%, respectively; reduced physical performance was regarded primarily as an outcome. The implication is straightforward: estimates of muscle quantity obtained from dual-energy X-ray absorptiometry (DXA) or bioelectrical impedance analysis (BIA) cannot substitute for direct assessment of muscle strength or physical performance. The complementary approaches used to characterize multidimensional muscle health in T2DM, including assessments of muscle quantity, strength, physical performance, and muscle composition, are summarized in **Table 2**.

**Table 2:** Complementary measures for multidimensional muscle–metabolic phenotyping in type 2 diabetes mellitus.

Assessment domain	Representative measures	Information captured	Relevance in T2DM	Main limitations/cautions
Screening	SARC-F (27)	Symptom- and function-based risk screening before objective testing.	Rapid, low-burden case finding in routine care.	Screening only; does not directly quantify muscle mass, strength, or tissue composition.
Muscle quantity	DXA: appendicular lean mass; BIA: skeletal muscle mass/skeletal muscle index (12, 28)	Muscle or lean-tissue quantity, usually normalized to body size.	Central to conventional sarcopenia frameworks and feasible for serial assessment.	DXA/BIA do not characterize tissue composition; BIA is hydration-sensitive; normalization method can change case classification.
Muscle strength	Handgrip and other direct strength tests (12, 26)	Force-generating capacity and a functional dimension of muscle health.	Strength deficits in T2DM may be more consistent than differences in muscle mass; low strength is required with low mass in AWGS 2025.	Effort- and technique-dependent; can be influenced by neuropathy, pain, arthritis, and other comorbidities.
Physical performance	Gait- and task-based functional performance tests (12, 26)	Integrated mobility and the clinical consequences of impaired muscle function.	Useful for characterizing functional impact and treatment-relevant outcomes.	Influenced by cardiovascular, neurologic, and musculoskeletal disease; AWGS 2025 places greater emphasis on performance as an outcome.
Computed tomography	Muscle area/index; attenuation or density; myosteatosis (11, 28–30)	Muscle quantity plus radiodensity as an imaging marker related to lipid infiltration.	Can reveal lower muscle density despite preserved muscle index and can use clinically acquired CT scans.	Ionizing radiation; slice level and thresholds vary; most T2DM evidence is observational and cross-sectional.
Magnetic resonance imaging	Muscle volume and fat infiltration (17, 28)	High-resolution assessment of muscle quantity and composition.	Can distinguish changes in muscle volume from changes in intramuscular fat during weight loss.	Cost and availability; protocol harmonization is limited; functional outcomes are not routinely integrated.
Muscle ultrasound	Muscle thickness/architecture and echogenicity-related measures (28)	Repeatable structural information without ionizing radiation.	Potentially scalable for serial bedside or outpatient assessment.	Operator and device dependence; limited standardization and validated prognostic thresholds.
BIA-derived phase angle	Phase angle (PhA) (31)	Electrical marker related to cell integrity and nutritional/hydration state.	Lower PhA has been associated with lower muscle mass and strength in T2DM and is easy to repeat.	Not equivalent to CT/MRI-defined myosteatosis or muscle density; hydration status affects interpretation.
Emerging metabolic research phenotype	HbA1c plus CGM-derived TIR, CV, MAGE, and TBR (13–16)	Average and dynamic glycemic exposure rather than muscle structure itself.	Exploratory research into associations between dynamic glycemic exposure and muscle phenotypes beyond HbA1c.	Not sarcopenia diagnostic measures; evidence is mainly observational, with residual confounding and reverse causation possible.

BIA, bioelectrical impedance analysis; CGM, continuous glucose monitoring; CT, computed tomography; CV, coefficient of variation; DXA, dual-energy X-ray absorptiometry; MAGE, mean amplitude of glycemic excursions; MRI, magnetic resonance imaging; PhA, phase angle; TBR, time below range; TIR, time in range.

AWGS 2025 further revised the diagnostic framework for Asian populations. Chen et al. (2025) (12) extended the applicable age range to adults aged 50–64 years and defined sarcopenia by the coexistence of low muscle mass and low muscle strength, while physical performance was positioned primarily as an outcome measure. This diagnostic structure should not be generalized across all consensus frameworks, because the roles assigned to muscle quantity, muscle strength, and physical performance differ among AWGS, EWGSOP, and other contemporary approaches (3–6, 26). Relative to earlier AWGS frameworks centered on case finding in older adults (5, 6), AWGS 2025 adopts a broader life-course perspective on muscle health and explicitly recognizes interactions between skeletal muscle and adipose tissue, bone, the brain, and the immune system.

The choice of functional test represents an additional source of diagnostic variability. In 100 community-dwelling older adults with T2DM, Barak et al. (2026) found that the prevalence of impaired muscle function varied markedly according to the assessment used, ranging from 1% with handgrip strength to 92% with the chair-stand test; maximal knee-extension strength provided an additional lower-extremity strength measure. These findings should not be interpreted as indicating that one test is universally superior to another, because the tests capture different aspects of muscle function and may be influenced by different clinical factors. Rather, they illustrate that the choice of functional assessment can substantially affect case identification and complicate comparisons across studies using different diagnostic procedures (32).

In T2DM, these changes in age eligibility and muscle-mass normalization can materially alter case identification. Hashimoto et al. (2026) (33) compared AWGS 2019 with AWGS 2025 in 754 Japanese participants with T2DM from the KAMOGAWA-A cohort. Sarcopenia prevalence increased from 15.0% to 23.6%, with most newly identified cases occurring in individuals aged <65 years or with a BMI ≥23 kg/m² who met the low ASM/BMI criterion. Sarcopenia defined by either framework was associated with DKD; fully adjusted ORs were 2.25 (95% CI 1.31–3.88) for AWGS 2019 and 1.78 (95% CI 1.17–2.70) for AWGS 2025. These findings show that the revised criteria broaden case detection, but they do not establish superior diagnostic accuracy. Whether the additional cases carry higher long-term risks of disability, cardiovascular disease, or death remains to be tested prospectively.

Terminological discipline is equally important. Throughout this review, muscle quantity refers to the amount of skeletal muscle or lean tissue, whereas muscle strength refers to force-generating capacity. Physical performance is a broader functional construct reflecting the integrated ability to perform mobility-related tasks and may be influenced by muscle strength as well as neurologic, musculoskeletal, cardiorespiratory, and other factors. Muscle composition refers to structural characteristics of muscle tissue, including fat infiltration and radiodensity. By contrast, “muscle quality” lacks a universally accepted operational definition and has been represented by distinct measures, including strength relative to muscle mass and imaging-derived characteristics of muscle composition. These constructs are related but should not be used interchangeably.

Accordingly, sarcopenia, isolated low muscle mass, lean-mass loss, dynapenia, and myosteatosis should also be distinguished. A reduction in lean mass during weight loss represents a change in body composition and, by itself, does not constitute a diagnosis of sarcopenia. This distinction is particularly important in T2DM, in which muscle quantity may be relatively preserved despite abnormalities in strength, physical performance, or muscle composition.

### 3.2 Epidemiologic burden of sarcopenia in T2DM

The epidemiologic association between T2DM and sarcopenia is supported by a relatively consistent body of evidence (34). In their meta-analysis of 15 studies, Anagnostis et al. (2020) (7) found 55% higher odds of sarcopenia among individuals with T2DM than among those with normal glucose metabolism (OR 1.55, 95% CI 1.25–1.91; I² = 34.6%); the association persisted in age- and sex-matched analyses. Notably, deficits in muscle strength and physical performance were more consistent than differences in muscle mass. This pattern again suggests that defining diabetes-related muscle impairment solely by low muscle mass is incomplete.

Evidence from Asian populations is broadly concordant. Wu et al. (2026) (8) synthesized 43 observational studies and reported study-specific prevalence estimates ranging from 4% to 50%, with a pooled prevalence of 17% (95% CI 14%–21%). Prevalence was 13% (95% CI 8%–18%) in middle-aged adults and 17% (95% CI 14%–21%) in older adults, with no statistically significant difference between the age groups; the estimate reached 28% in Southeast Asia. An earlier meta-analysis by Feng et al. (2022) (35) estimated an overall prevalence of 18% (95% CI 16%–20%) among patients with diabetes.

These pooled figures should not obscure substantial methodological heterogeneity. AWGS, EWGSOP, and other frameworks differ in diagnostic algorithms, component definitions, and cutoffs (3–6), while the choice of functional assessment can further alter case identification because handgrip strength, lower-extremity strength, and chair-stand performance capture related but non-equivalent aspects of muscle function (32). Methods used to quantify muscle quantity also differ: DXA and BIA have distinct measurement properties, and appendicular muscle mass may be normalized to height, body weight, or BMI. Population characteristics, including age, sex, ethnicity, adiposity, diabetes duration, comorbidity burden, and care setting, provide additional sources of variation. Accordingly, pooled prevalence estimates of approximately 17%–18% should be interpreted as population-level benchmarks rather than fixed values applicable to every T2DM population.

### 3.3 Clinical significance and high-risk phenotypes

The clinical importance of sarcopenia in T2DM rests primarily on its association with adverse outcomes (36). In a systematic review and meta-analysis of 15 studies, Bai et al. (2025) (9) found that sarcopenia in T2DM was associated with higher all-cause mortality (HR 1.72, 95% CI 1.28–2.32) and a higher risk of cardiovascular disease (HR 1.94, 95% CI 1.67–2.25). Pooled analyses of diabetes-related complications also suggested higher risk (HR 1.12, 95% CI 1.09–1.15; OR 2.49, 95% CI 1.53–4.05). Sarcopenia may therefore serve as a clinically informative phenotype that integrates age, metabolic status, nutritional state, inflammatory burden, and chronic complications.

These associations should not be interpreted as proof of causality. Sarcopenia frequently coexists with older age, longer diabetes duration, chronic kidney disease, malnutrition, and low physical activity. Even carefully adjusted observational models cannot fully eliminate residual confounding. The more defensible interpretation is that sarcopenia identifies a high-risk subgroup within T2DM; whether sarcopenia independently causes cardiovascular events or mortality requires stronger longitudinal and interventional evidence.

Evidence on associated factors is similarly dominated by observational studies. In the Asian meta-analysis by Wu et al. (2026) (8), older age (OR 1.12), higher HbA1c (OR 1.11), DKD (OR 1.76), and peripheral neuropathy (OR 3.28) were associated with greater odds of sarcopenia, whereas better nutritional status, assessed with the Mini Nutritional Assessment, was associated with lower odds (OR 0.37). Feng et al. (2022) additionally (35) identified age, HbA1c, visceral fat area, diabetes duration, DKD, and high-sensitivity C-reactive protein as correlates. Because most contributing studies lacked a temporal sequence, these variables are better described as correlates or high-risk features than as established causal determinants.

The relationship between BMI and sarcopenia is particularly easy to misinterpret. Wu et al. (2026) (8) found that higher BMI was associated with lower odds of sarcopenia (OR 0.66), whereas higher body-fat percentage was associated with greater odds in both men and women (ORs 1.26 and 1.27, respectively). BMI does not distinguish adipose tissue from skeletal muscle, nor does it capture intramuscular fat. In addition, case classification can be affected by body size when relative muscle-mass indices are used. Thus, the apparent “protective” association of higher BMI in some models should not be construed as evidence that obesity protects skeletal muscle.

## 4 Multidimensional muscle phenotypes in T2DM

### 4.1 Muscle quantity, strength, and physical performance

Early studies in T2DM already suggested that absolute muscle mass is an imperfect proxy for muscle function (37, 38). In the Health ABC Study, Park et al. (2006) (10) compared 485 older adults with diabetes with 2,133 without diabetes. Participants with diabetes had greater appendicular muscle mass because of their larger body size, yet men had lower strength and women did not exhibit the strength expected from their greater muscle mass. When muscle-specific strength was expressed as force per unit of regional muscle mass—a measure described as “muscle quality” in the original study—both men and women with diabetes had poorer values. The deficit was more pronounced among individuals with diabetes duration ≥6 years or HbA1c >8%.

Three-year follow-up in the same cohort strengthened this observation. Compared with participants without diabetes, those with T2DM experienced a greater decline in leg muscle mass (-0.29 ± 0.03 vs. -0.23 ± 0.01 kg, P < 0.05), a larger loss of leg strength (-16.5 ± 1.2 vs. -12.4 ± 0.5 Nm, P = 0.001), and a greater reduction in strength per unit of muscle mass (-1.6 ± 0.2 vs. -1.2 ± 0.1 Nm/kg, P < 0.05) (39). Together with the meta-analysis by Anagnostis et al. (2020) (7), these findings demonstrate that muscle quantity, absolute strength, and strength relative to muscle mass represent related but non-equivalent dimensions of muscle health in T2DM.

### 4.2 Muscle composition, muscle quality, and myosteatosis

Imaging studies have identified alterations in muscle composition in T2DM that may occur despite relatively preserved muscle quantity. Lower CT attenuation generally indicates greater lipid content within muscle (29), whereas MRI can characterize muscle volume and fat infiltration in greater detail (40). These measures provide complementary information on muscle composition but should not be regarded as interchangeable indicators of a single construct.

Kim et al. (2022) (30) conducted a cross-sectional analysis of 20,986 adults undergoing abdominal CT. Participants with T2DM did not have less total abdominal or skeletal muscle area; however, they had less normal-attenuation muscle area (NAMA) and a lower NAMA-to-total abdominal muscle area (TAMA) ratio. From the lowest to the highest quartile of the NAMA/TAMA index, T2DM prevalence declined from 19.3% to 9.5% in men and from 12.3% to 3.0% in women; multivariable-adjusted ORs comparing the highest with the lowest quartile were 0.75 and 0.68, respectively. Zhang et al. (2025) (11) reported a similar pattern in 1,225 adults aged ≥65 years: T2DM was associated with lower T4-level skeletal muscle density but not with skeletal muscle index. HbA1c was inversely correlated with muscle density (ρ = -0.194, P < 0.05), and the association between T2DM and lower muscle density persisted after multivariable adjustment (β = -0.069, P = 0.002).

Although these studies used different CT levels and muscle metrics, they converge on an important observation: the association between T2DM and abnormal muscle composition or density may be more consistent than the association with absolute muscle quantity. This provides clinical support for myosteatosis as a potentially relevant phenotype of diabetes-related muscle impairment.

Current imaging evidence, however, is insufficient to establish myosteatosis as an obligatory intermediate step between T2DM and sarcopenia. Most studies are cross-sectional and cannot determine whether intramuscular fat accumulation is a consequence of metabolic dysfunction or whether impaired muscle composition contributes to insulin resistance and the development of T2DM. Definitions of myosteatosis, CT thresholds, anatomic sites, and normalization strategies also remain inconsistent (41). At present, myosteatosis is best viewed as a candidate metabolic phenotype linking ectopic lipid deposition, insulin resistance, and muscle dysfunction; its causal role and independent prognostic value require longitudinal validation.

## 5 Pathophysiological mechanisms linking T2DM and muscle impairment

Muscle impairment in T2DM likely arises from interacting mechanisms rather than a single pathway (2, 42). Insulin resistance, chronic hyperglycemia, inflammation, oxidative stress, lipotoxicity, and diabetes-related complications can affect skeletal muscle through partially overlapping pathways involving impaired anabolic signaling, increased proteolysis, mitochondrial dysfunction, ectopic lipid accumulation, and neuromuscular impairment. These processes need not affect all dimensions of muscle health equally: some may primarily alter muscle composition or metabolic capacity before substantial loss of muscle quantity becomes apparent, whereas neuropathy and disuse may have more immediate consequences for strength and physical performance. Conversely, skeletal muscle is a major site of peripheral glucose disposal, and reductions in muscle metabolic capacity, strength, or physical activity may further impair glucose handling. This provides a biological basis for bidirectional feedback between metabolic dysfunction and declining muscle health and helps explain why T2DM may be associated with impaired muscle composition or function even when absolute muscle quantity is relatively preserved.

### 5.1 Insulin resistance and metabolic dysregulation

Insulin resistance is a central component of this network (1, 43). Under physiological conditions, insulin promotes skeletal-muscle glucose uptake and supports protein anabolism while suppressing proteolysis through pathways that include IRS-1/PI3K/Akt signaling. In T2DM, impaired insulin signaling reduces metabolic responsiveness and may attenuate anabolic signaling while relieving inhibition of FoxO-mediated protein degradation. These abnormalities can create an unfavorable balance between muscle protein synthesis and breakdown, particularly when combined with physical inactivity or inadequate nutritional intake. Age-related anabolic resistance may further reduce the skeletal-muscle response to dietary protein and resistance exercise, providing a potential explanation for the heightened vulnerability of older adults with T2DM. Importantly, metabolic dysfunction may precede overt loss of muscle mass, reinforcing the need to assess strength and function rather than relying on muscle quantity alone.

### 5.2 Hyperglycemia, AGEs, inflammation, and oxidative stress

AGEs offer another mechanistic link between chronic hyperglycemia and muscle dysfunction. A systematic review by Granic et al. (2023) (44), comprising 14 cross-sectional studies and one prospective study, found that higher skin autofluorescence or circulating AGE concentrations were generally associated with poorer muscle strength, lower muscle mass, and worse physical performance; the human evidence, however, remains predominantly associative. Mechanistic support comes from the animal study by Du et al. (2023) (45), in which AGE exposure increased reactive oxygen species and endoplasmic reticulum stress, activated PERK/FOXO1 signaling, and upregulated MAFbx. These changes coincided with smaller muscle-fiber cross-sectional area, fibrosis, intramuscular lipid accumulation, and insulin resistance; FOXO1 silencing partially attenuated the phenotype. The AGE–oxidative/endoplasmic-reticulum-stress–FOXO1 axis is therefore biologically plausible, but its causal contribution to sarcopenia in human T2DM remains unproven.

### 5.3 Lipotoxicity and ectopic fat

Lipotoxicity may also contribute to altered muscle composition in T2DM. Increased circulating free fatty acids and limited adipose-tissue storage capacity can promote ectopic lipid deposition in skeletal muscle. Intramuscular lipids and their metabolites may in turn disrupt mitochondrial oxidation and insulin signaling while amplifying inflammation and oxidative stress. Myosteatosis may therefore both reflect cumulative metabolic exposure and participate in skeletal-muscle insulin resistance. This framework helps explain why some individuals with obesity and T2DM have preserved absolute muscle mass yet exhibit impaired strength or muscle quality.

### 5.4 Diabetes-related complications

Diabetes-related complications may further accelerate functional decline. DPN can impair contractile capacity through motor-nerve injury, denervation, and loss of motor units; pain, balance impairment, and reduced activity may add a component of disuse-related muscle loss. Renal disease provides a second example of organ–muscle interaction. In a hospital-based prospective cohort, Wu et al. (2026) (46) separately analyzed 4,826 participants without DKD at baseline and 4,798 without sarcopenia at baseline. Sarcopenia was associated with incident DKD (adjusted HR 1.587, 95% CI 1.432–1.755), whereas baseline DKD was associated with incident sarcopenia (adjusted HR 2.036, 95% CI 1.741–2.384). Although unmeasured confounding cannot be excluded, the temporal pattern supports a potentially bidirectional kidney–muscle relationship. Taken together, sarcopenia in T2DM is better understood as a multifactorial clinical phenotype than as an isolated complication driven by a single molecular pathway.

## 6 Multidimensional assessment of muscle health

### 6.1 Complementary assessment of muscle quantity, strength, physical performance, and composition

A multidimensional assessment of muscle health requires distinction between the construct of interest and the method used to measure it. Muscle quantity describes the amount of muscle tissue; muscle strength reflects force-generating capacity; physical performance captures integrated task-level function; and muscle composition describes structural characteristics such as fat infiltration and radiodensity. No single assessment modality captures all of these dimensions.

Conventional sarcopenia assessment combines measures of muscle quantity with direct assessment of muscle strength and, depending on the diagnostic framework, physical performance (12, 26, 27). DXA is widely used to quantify appendicular lean tissue and has good reproducibility, whereas BIA is less expensive and more accessible but is more sensitive to hydration status and prediction equations. Neither method directly characterizes intramuscular fat or muscle architecture. Handgrip and other direct strength tests provide information on force-generating capacity, while gait- and task-based tests capture broader physical performance. These functional measures are inexpensive and clinically feasible, but their interpretation may be affected by effort, technique, neuropathy, pain, joint disease, and other comorbidities.

CT and MRI provide more detailed characterization of muscle morphology and composition. CT can quantify muscle cross-sectional area and attenuation, allowing muscle quantity and radiodensity-related features of myosteatosis to be assessed simultaneously; clinically acquired CT images may also permit opportunistic muscle evaluation (11, 28–30). MRI provides high-resolution assessment of muscle volume and fat infiltration without ionizing radiation and can distinguish changes in muscle volume from changes in intramuscular fat (17, 28, 40). However, CT entails radiation exposure when performed specifically for muscle assessment, whereas MRI is limited by cost, examination time, availability, and protocol complexity. These constraints reduce their practicality for repeated routine muscle assessment despite the richer compositional information they provide.

Muscle ultrasound offers a more accessible and repeatable approach to structural muscle assessment. It can quantify muscle thickness and cross-sectional dimensions, characterize architectural features such as fascicle length and pennation angle, and evaluate echogenicity as an indirect marker of changes in muscle composition (28). Compared with CT and MRI, ultrasound is radiation-free, portable, relatively inexpensive, and suitable for repeated bedside or outpatient measurements, making it attractive for longitudinal assessment and settings in which advanced imaging is not readily available. Its limitations are equally important: image acquisition and analysis are operator dependent, measurements can be influenced by probe position, orientation, and compression, and protocols differ across muscles, devices, and analytic methods. Echogenicity also does not provide the same direct compositional information as CT attenuation or MRI-based fat quantification. Thus, although ultrasound may complement conventional muscle assessment, greater protocol standardization, reproducibility testing, and validation of clinically meaningful thresholds are needed before measurements can be compared reliably across studies or incorporated broadly into routine sarcopenia assessment (28).

BIA-derived phase angle (PhA) provides a different type of complementary information. PhA is an electrical marker related to cell integrity and nutritional or hydration status, and lower PhA has been associated with lower muscle mass and strength in patients with T2DM (31). It is inexpensive and repeatable, but hydration status affects its interpretation, and PhA should not be treated as equivalent to CT- or MRI-defined myosteatosis, muscle density, or other direct measures of muscle composition.

These approaches are therefore complementary rather than interchangeable. In practice, the choice of assessment should be guided by the clinical or research question, required muscle domain, availability of equipment and expertise, feasibility of repeated measurement, and patient characteristics. DXA or BIA can provide measures of muscle quantity, strength and performance tests characterize functional dimensions, and CT, MRI, or ultrasound can provide additional structural or compositional information at different levels of detail and resource intensity. A layered approach may be particularly informative in T2DM because reliance on muscle quantity alone can miss individuals with relatively preserved muscle mass but impaired strength, physical performance, or muscle composition.

### 6.2 Continuous glucose monitoring as an emerging research tool

CGM is not a diagnostic or established assessment modality for sarcopenia. Rather, it is an emerging research tool for characterizing dynamic glycemic exposure and investigating its relationship with muscle phenotypes in T2DM. Unlike HbA1c, which summarizes average glycemia over time, CGM captures within-day glycemic patterns through metrics such as time in range (TIR), coefficient of variation (CV), mean amplitude of glycemic excursions (MAGE), and time below range (TBR) (13, 14). These metrics may provide complementary metabolic context when studying muscle health, but their clinical role in sarcopenia assessment has not been established.

Direct clinical evidence linking CGM-derived metrics to muscle outcomes remains limited and is based primarily on observational studies. In 310 hospitalized adults aged ≥60 years with T2DM, Duan et al. (2026) (15) reported sarcopenia in 32.3% of participants according to AWGS 2019 criteria (6). Each 10-percentage-point increase in TIR was associated with lower odds of sarcopenia (OR 0.83, 95% CI 0.74–0.93), and TIR was associated with skeletal muscle index and grip strength, but not with physical performance. In another study of 303 older adults with T2DM, CGM metrics were examined alongside chest CT measures; greater glycemic variability, including higher CV, was associated with a lower pectoralis muscle index (16).

These findings support a potential association between dynamic glycemic exposure and muscle phenotype, but they do not establish CGM as a clinical tool for diagnosing, screening, or monitoring sarcopenia. The available studies are observational, and residual confounding and reverse causation remain important concerns: individuals with better physical function, higher activity, or better nutritional status may also have more stable glycemia. Moreover, the available evidence does not establish that increasing TIR or reducing glycemic variability prevents or reverses sarcopenia. Prospective studies should determine whether CGM-derived metrics independently predict longitudinal changes in muscle quantity, strength, physical performance, or composition beyond conventional glycemic measures such as HbA1c. Intervention studies would then be required to establish whether modifying glycemic variability improves muscle-related outcomes. Until such evidence is available, CGM should be regarded as an emerging research tool for muscle–metabolic phenotyping rather than a component of routine sarcopenia assessment.

## 7 Sarcopenic obesity

Sarcopenic obesity (SO) is a particularly important phenotype in T2DM because excess body weight can obscure low muscle reserve or impaired muscle function (47, 48). Obesity and sarcopenia are not mutually exclusive: an individual may have a high BMI or substantial total lean mass while still having insufficient muscle relative to body size, reduced strength, or pronounced intramuscular fat infiltration. Normal or elevated body weight therefore does not rule out poor muscle health.

The evaluation of SO requires assessment of both adiposity and sarcopenia rather than reliance on BMI alone. The ESPEN/EASO consensus proposes a two-step approach consisting of screening followed by diagnosis (47). In individuals with elevated BMI or waist circumference and clinical risk factors or suspicion of sarcopenia, diagnostic assessment combines evidence of impaired skeletal muscle function with altered body composition. Muscle function can be assessed using measures such as handgrip strength or the chair-stand test, whereas body composition can be evaluated using DXA or BIA to characterize adiposity and muscle mass (47). Importantly, the interpretation of muscle mass in obesity requires consideration of body size, because absolute muscle mass may appear preserved or elevated in individuals with excess body weight despite inadequate muscle relative to body size. Thus, SO cannot be identified reliably from BMI, body weight, or absolute lean mass alone.

A systematic review and meta-analysis by Zhou et al. (2023) (49) included 20 studies and estimated an SO prevalence of approximately 27% among patients with diabetes using a random-effects model. However, case identification varied across studies because SO prevalence depends on how both components of the phenotype are operationalized. Obesity may be defined using BMI, waist-based measures, or body-fat measures, whereas sarcopenia definitions may differ in the muscle quantity, strength, and functional criteria applied, as well as in measurement methods and cutoffs (47–49). Consequently, the same population may yield different prevalence estimates under different diagnostic combinations, limiting direct comparison across studies. The pooled estimate of approximately 27% should therefore be interpreted as an average across heterogeneous definitions and populations rather than as a fixed prevalence applicable to all patients with T2DM. Narrative synthesis has also linked SO with lower estimated glomerular filtration rate, overt proteinuria, cognitive impairment, and insulin resistance (49), but these observational associations do not establish causality.

SO also helps reconcile the apparently paradoxical relationship between BMI and sarcopenia. Higher BMI may reduce the likelihood of meeting conventional low-muscle-mass thresholds, yet greater body weight does not imply better muscle health once central adiposity, intramuscular fat, and strength are considered. As weight reduction becomes a central therapeutic objective in T2DM, the clinical goal should be refined from weight loss alone to preferential loss of adipose tissue with preservation of strength and function, particularly in patients with low baseline muscle reserve or impaired muscle composition.

## 8 Therapeutic implications

### 8.1 Exercise and nutrition as the foundation of muscle preservation

Among available interventions, resistance training has the most direct evidence for improving both muscular and metabolic outcomes in T2DM (50). Wang et al. (2025) (18) pooled 43 randomized controlled trials involving 2,012 adults aged ≥50 years with T2DM. Compared with control conditions, resistance training reduced HbA1c by 0.55 percentage points, lowered HOMA-IR by 1.15 and fasting glucose by 6.99 mg/dL, and increased muscle mass by 0.89 kg. Upper- and lower-extremity strength improved substantially (SMD 2.28 and 2.02, respectively). C-reactive protein decreased (SMD -0.80), whereas TNF-α and IL-6 did not change significantly. Overall, resistance training provides a strong foundation for muscle-preserving care in T2DM because it improves metabolic control and muscle-related outcomes concurrently. These findings support resistance training as a core component of management, but evidence of efficacy should be distinguished from the practical prescription of exercise, which requires specification of training frequency, intensity, volume, and progression.

For clinical implementation, resistance exercise should be prescribed progressively rather than simply recommended as a general increase in physical activity. A practical target is at least two resistance-training sessions per week involving the major muscle groups, with approximately 8–10 exercises performed for 2–4 sets of 8–10 repetitions at moderate-to-vigorous intensity; loads permitting only 8–10 repetitions correspond approximately to 70%–84% of one-repetition maximum (1RM) (51). Training volume and resistance should be increased progressively according to individual capacity and tolerance, with appropriate consideration of diabetes-related complications and other comorbidities (51). In older or deconditioned adults, initial exercise intensity and volume should be individualized and progressed as technique, tolerance, and strength improve.

Functional gains from exercise do not always track changes in muscle mass. Trials that rely only on skeletal muscle index or lean mass may therefore underestimate improvements in strength, mobility, and metabolic adaptation. Exercise studies should, where feasible, report muscle mass, muscle strength, and physical performance together.

Nutritional strategies should complement resistance exercise rather than be considered in isolation. For generally healthy older adults, a daily protein intake of approximately 1.0–1.2 g/kg body weight has been recommended to support muscle health, with higher intakes potentially required in the presence of acute or chronic illness, depending on clinical circumstances (19). Protein intake should be distributed across meals and combined with resistance exercise to provide both nutritional substrate and an anabolic stimulus. However, these targets should not be applied uniformly to all patients with T2DM. In individuals with chronic kidney disease, protein intake should be individualized according to kidney function, nutritional status, and renal dietary guidance. In patients with obesity or those undergoing intentional weight loss, adequate protein intake and resistance exercise should be incorporated into the weight-management strategy to reduce the risk of disproportionate loss of lean tissue while promoting preferential loss of adipose tissue. Accordingly, muscle-preserving care in T2DM should pair progressive resistance exercise with individualized nutritional support, while monitoring changes in muscle strength and physical performance rather than body weight or lean mass alone.

### 8.2 SGLT2 inhibitors: interpreting changes in body composition

Studies of sodium-glucose cotransporter 2 (SGLT2) inhibitors illustrate the distinction between changes in body composition and sarcopenia. In a meta-analysis of 25 randomized controlled trials involving 2,286 participants with T2DM, Zhang et al. (2023) (20) found that SGLT2 inhibitors reduced body weight and fat mass but were also associated with a mean reduction in lean mass of 0.80 kg (95% CI -1.43 to -0.16), a reduction in skeletal muscle mass of 0.38 kg (95% CI -0.65 to -0.10), and a small decline in skeletal muscle index. Body water decreased by 0.96 kg (95% CI -1.68 to -0.23). These findings indicate that body composition changes during treatment, but they do not establish drug-induced sarcopenia.

At least three considerations qualify the interpretation. First, most trials measured lean mass or muscle mass rather than sarcopenia defined by AWGS or EWGSOP criteria. Second, fluid loss with SGLT2 inhibition may influence BIA-derived estimates of lean tissue. Third, data on muscle strength and physical performance remain sparse. The evidence therefore supports monitoring muscle status during treatment but does not support the conclusion that SGLT2 inhibitors cause sarcopenia.

### 8.3 GLP-1 receptor agonists: clinical meaning of lean-mass loss during weight reduction

Concern about GLP-1 receptor agonists largely reflects the loss of lean mass that accompanies substantial weight reduction (22–24). As these agents are used more widely for T2DM and obesity, it becomes increasingly important to determine whether older adults, frail individuals, or patients with limited baseline muscle reserve experience clinically meaningful muscle loss.

Current human evidence neither demonstrates that GLP-1 receptor agonists directly damage skeletal muscle nor establishes a clear protective effect against sarcopenia. The systematic review and meta-analysis by Chen et al. (2025) (21) included 18 studies: six human studies reported lean mass, four clinical trials reported skeletal muscle index, and eight studies were conducted in animals. Preclinical data suggest potential effects on myogenic signaling, inflammation, and muscle metabolism, but human evidence on strength, physical performance, and rigorously defined sarcopenia remains limited. Large trials with prespecified functional and clinical muscle outcomes are therefore required.

The clinically relevant question is not simply whether lean mass declines during GLP-1 receptor agonist therapy. Rather, the decline must be interpreted relative to the magnitude of weight loss and in conjunction with changes in muscle strength, physical performance, and tissue composition. Only this multidimensional assessment can determine whether a change in lean mass represents clinically meaningful muscle deterioration.

### 8.4 Tirzepatide and SURPASS-3 MRI: muscle volume versus muscle composition

The SURPASS-3 MRI analysis provides a more granular view of this issue. Sattar et al. (2025) (17) conducted a *post hoc* analysis of the MRI substudy of SURPASS-3, a randomized, open-label, parallel-group phase 3 trial. Of 296 participants enrolled in the MRI substudy, 246 had evaluable MRI data at 52 weeks. In the pooled tirzepatide groups, muscle fat infiltration declined by 0.36 percentage points (95% CI -0.48 to -0.25), thigh muscle volume decreased by 0.64 L (95% CI -0.74 to -0.54), and the muscle-volume Z score declined by 0.22. The observed change in muscle volume was close to that predicted from the magnitude of weight loss (difference -0.04 L, 95% CI -0.11 to 0.03; P = 0.22), whereas the reduction in muscle fat infiltration exceeded that predicted from weight change alone (difference -0.42 percentage points, 95% CI -0.54 to -0.31; P < 0.0001).

These findings show why absolute loss of muscle volume during substantial weight reduction must be interpreted together with muscle composition and function. If muscle volume changes approximately as expected for the degree of weight loss while intramuscular fat decreases, muscle-volume loss alone is an insufficient basis for inferring treatment-related muscle injury.

SURPASS-3 MRI nevertheless does not demonstrate that tirzepatide prevents sarcopenia. It was a *post hoc* MRI substudy of a randomized phase 3 trial; sarcopenia, muscle strength, and physical performance were not primary outcomes, and the study was industry funded. Its principal contribution is methodological: trials of pharmacologic weight loss should evaluate muscle quantity, composition, and function together rather than treating a single change in muscle volume as synonymous with muscle health.

Future trials of potent weight-loss therapies should therefore move beyond the question of how much lean mass is lost. A meaningful assessment should determine whether fat mass, muscle mass, intramuscular fat, muscle strength, and physical performance change in parallel or diverge. Body-composition endpoints need to be paired with functional muscle outcomes to estimate the net effect of treatment on muscle health.

## 9 Discussion and future perspectives

The first major limitation of the current evidence base is the lack of harmonized definitions and measurements. Studies apply different diagnostic frameworks (3–6), muscle-mass normalization approaches, and measurement techniques, while proposed indices of “muscle quality” capture distinct biological constructs. This heterogeneity limits cross-study comparisons of prevalence, weakens risk stratification, and complicates the interpretation of pooled estimates.

The second major limitation is causal inference. Research on myosteatosis, AGEs, TIR, and glycemic variability is still dominated by cross-sectional or observational designs, making temporal ordering uncertain. Contemporary pharmacologic trials provide extensive data on body weight and body composition, yet sarcopenia, strength, physical performance, and long-term clinical outcomes are rarely prespecified endpoints. Many proposed measures should therefore remain classified as candidate risk markers or mechanistic signals rather than being translated prematurely into diagnostic thresholds or therapeutic targets.

Future work should prioritize three objectives. First, long-term prospective cohorts should enroll middle-aged as well as older adults with T2DM under harmonized diagnostic criteria and characterize the natural history of muscle mass, strength, composition, and physical performance in relation to mortality, cardiovascular events, falls, and DKD. Second, the field needs a core outcome set for muscle health in T2DM and should test whether myosteatosis, PhA, and CGM-derived metrics provide incremental prognostic information beyond conventional clinical variables. Third, trials of potent weight-loss therapies, including GLP-1 receptor agonists and tirzepatide, should prespecify muscle-centered outcomes and examine whether resistance training and individualized nutrition preserve strength and function during fat loss. Multimodal phenotyping will become clinically useful only after its components have been standardized and validated longitudinally.

## 10 Conclusion

Current evidence indicates that individuals with T2DM have higher odds of sarcopenia than those with normal glucose metabolism and that sarcopenia in T2DM is associated with higher risks of mortality, cardiovascular disease, and diabetes-related complications (7, 9). Older age, longer diabetes duration, higher HbA1c, poorer nutritional status, DKD, and peripheral neuropathy are associated with greater odds of sarcopenia (8, 35). Sarcopenia is therefore an informative clinical phenotype for comprehensive risk assessment in T2DM, although its independent causal role remains uncertain.

Diabetes-related muscle impairment is not reducible to loss of muscle mass. The Health ABC studies, meta-analytic evidence, and more recent CT studies show that lower strength, reduced strength per unit of muscle, lower muscle density, and greater fat infiltration can occur even when differences in muscle quantity are small or absent (7, 10, 11, 30, 39). Insulin resistance, AGE-related stress, lipotoxicity, chronic inflammation, and neurologic and renal complications may jointly contribute to these abnormalities. The GLIS framework and AWGS 2025 further support a multidimensional view of muscle health by emphasizing strength, muscle-specific strength, and life-course assessment (12, 26).

In clinical assessment and intervention research, DXA/BIA, CT/MRI, ultrasound, PhA, and CGM provide complementary information on muscle quantity, tissue composition, cellular state, or dynamic glycemic exposure, but these measures have not yet been integrated into a validated predictive framework. Resistance training has relatively direct randomized-trial evidence for improving both metabolic and muscle outcomes. In contrast, reductions in lean mass or muscle volume during treatment with SGLT2 inhibitors, GLP-1 receptor agonists, or tirzepatide must be interpreted alongside muscle strength, physical performance, and composition (17, 18, 20, 21).

The field is moving from the detection of low muscle mass toward a more complete assessment of muscle health in T2DM. Progress will depend less on proliferating additional, non-comparable biomarkers than on harmonizing definitions and outcomes, establishing longitudinal evidence, and identifying which muscle phenotypes materially improve risk stratification and therapeutic decision-making. Only then can muscle health be incorporated coherently into long-term, comprehensive management of T2DM.
